# Effects of Sleep Fragmentation and Induced Mood on Pain Tolerance and Pain Sensitivity in Young Healthy Adults

**DOI:** 10.3389/fpsyg.2018.02089

**Published:** 2018-10-31

**Authors:** Ragna Rosseland, Ståle Pallesen, Inger Hilde Nordhus, Dagfinn Matre, Tone Blågestad

**Affiliations:** ^1^Department of Psychosocial Science, University of Bergen, Bergen, Norway; ^2^Norwegian Competence Center for Sleep Disorders, Haukeland University Hospital, Bergen, Norway; ^3^Department of Clinical Psychology, University of Bergen, Bergen, Norway; ^4^Department of Behavioural Sciences in Medicine, University of Oslo, Oslo, Norway; ^5^Department of Work Psychology and Physiology, National Institute of Occupational Health, Oslo, Norway

**Keywords:** sleep fragmentation, pain, mood induction, sleep, mood pain tolerance, pain sensitivity, mood elicitation

## Abstract

**Background:** Experimental research exploring the sleep/pain-relationship has typically focused on total or partial sleep deprivation, hereby failing to reproduce the mere fragmented sleep pattern typically observed in patients with chronic pain. Further, little research is done on *how* affect moderates the sleep–pain relationship after sleep fragmentation. The present study sought to clarify the relationship between pain, sleep and positive and negative affect.

**Methods:** In an experimental counterbalanced crossover design, 35 healthy young adults were subjected to several pain measures after one night of fragmented sleep, compared to one control night of normal sleep, both conducted in their own homes, and respectively, positive and negative affect induction using validated film clips and facial feedback procedures. Sleep was monitored using sleep diaries.

**Results:** Increased pain sensitivity after one night of experimentally induced sleep fragmentation was found, compared to after one control night of undisturbed sleep. No main effects of induced affect on pain were found, and sleep x induced affect interaction was not significant.

**Conclusion:** The present study supports the adverse effect of sleep fragmentation on pain sensitivity, however, affect was not found to be a moderator in the sleep–pain relationship. The results underline the need for further research within this field.

## Introduction

Sleep duration and sleep quality are both physiologically important for pain modulation in the central nervous system (e.g., Haack et al., 2012, pp. 522–533; Smith et al., 2007, pp. 494–505.). In line with this, disturbed sleep is found to increase pain sensitivity, and to decrease pain threshold in healthy individuals (e.g., Schrimpf et al., 2015, pp. 1313–1320; Lautenbacher et al., 2006, pp. 357–369; Onen et al., 2005, pp. 422–431). Previous studies have used an experimental model for studying the physiological changes in pain perception, using a single or a couple of nights with little or no sleep, implying total, or partial sleep deprivation (e.g., Onen et al., 2001; Haack et al., 2009), or sleep restriction (e.g., Haack and Mullington, 2005; Roehrs et al., 2006). However, such models fail to grasp the typical sleep problems characteristic of chronic pain patients, that is fragmented sleep, rather than total or partial sleep deprivation (Bjurstrom and Irwin, 2016, pp. 74–86). Common to all experiments utilizing sleep fragmentation is that they produce repeated bouts of wakefulness during the night and is as such disrupting sleep continuity (Van Someren et al., 2015, pp. 13889–13895). Studies may differ in number of forced awakenings or total amount of wakefulness during the night. Smith et al. (2007) were the first to conduct a study using sleep fragmentation and subsequently investigating its impact on pain processing, hereby creating a clinically relevant model of sleep fragmentation. A recent study (Iacovides et al., 2017) utilized sleep fragmentation as operationalized by Smith et al. (2007), whereby healthy participants were subject to two nights of a random sequence of one 60-min awakening, and several 20-min awakenings across the nights in order to study the sleep–pain relationship (Iacovides et al., 2017, pp. 844–854). The results showed that sleep fragmentation induced hyperalgesia to superficial and deep-muscle pain, loss of pain inhibition, and an increase in spontaneous pain in healthy women, thus supporting previous research using sleep restriction (Finan et al., 2013, pp. 1539–1552). However, Matre et al. (2016) found no decreased conditioned pain modulation (CPM) after partial sleep restriction (Matre et al., 2016, pp. 408–416); implying further research is needed in order to clarify this relationship.

Mood disturbance frequently co-occurs with pain and sleep complaints (Goodin et al., 2011, pp. 913–922), yet the role of mood as a moderator of the sleep–pain relationship has so far received less attention and is poorly understood. Sleep restriction has been shown to reduce general measures of emotional and physical well-being (Kahn et al., 2014, pp. 825–832). In turn, mood or affect modulates pain. Positive affect is psychometrically distinct from negative affect (Watson et al., 1988; Smith and Zautra, 2008, pp. 799–810), and positively valenced emotions generally inhibit pain, with greater inhibition resulting from greater positive arousal (Rhudy, 2016). Furthermore, positive affect has been shown to act as a protective factor in the relation between negative affect and chronic pain (Zautra et al., 2005, pp. 212–220), promoting resilient physical and psychosocial functioning among individuals suffering from chronic pain (Ong et al., 2010, pp. 516–523) and insomnia (Zohar et al., 2005, pp. 47–54). In contrast, negatively valenced emotions with low-to-moderate arousal are shown to increase pain, with greater facilitation resulting from greater negative arousal (Rhudy, 2016). Mood disturbance could thus potentially modulate pain processing as an integral part of the reciprocal sleep–pain relationship. (Vgontzas et al., 2008, pp. 1451–1459; Chung and Tso, 2010, pp. 752–758; Goodin et al., 2011, pp. 913–922). Supporting this, a study by Finan et al. (2016), found that chronic pain and sleep fragmentation impaired positive affective processes (e.g., anhedonia), and triggered negative affective processes (e.g., anger), or both (Finan et al., 2016). Here, one single night of sleep fragmentation was found to reduce perceived positive affect relative to a night of uninterrupted sleep (Finan et al., 2016, p. 6). Furthermore, sleep fragmentation weakened the inhibition of pain by positive affect, and also decreased positive affect (Finan et al., 2016). In order to further assess the role of positive or negative affect on pain modulation as related to sleep, experimental mood induction should be utilized, however, to the authors knowledge no such study is yet conducted.

The present study therefore aimed to clarify the relationship between pain, sleep and positive and negative affect. In order to extend current knowledge, a forced awakening experimental sleep paradigm was utilized, mimicking the sleep pattern typically observed in chronic pain patients. Furthermore, through the use of validated mood induction techniques, positive and negative mood was induced prior to experimental pain testing. Dependent measures included pain tolerance, perceived pain threshold, pain intensity, and pain inhibition measured using the cold pressor test (CPT) and pressure algometry. The present study utilizes a paradigm testing pain inhibiting called CPM (Yarnitsky et al., 2010). In the CPM-paradigm, the change in a perceived painful “test stimulus” induced by another painful “conditioning stimulus,” is an indicator of endogenous pain inhibition. Firstly, we hypothesized that sleep fragmentation would increase the perception of pain. More specifically, we expected that when sleep was fragmented, participants would show reduced pain tolerance, pain threshold and pain inhibition, and increased pain intensity compared to when subjected to experimentally undisturbed sleep. It was further hypothesized that positive affect would attenuate, and that negative affect would amplify pain perception, respectively. Finally, we also expected an interaction effect to be present between sleep and mood on pain.

## Materials and Methods

### Participants

This study used a sample recruited based on convenience, reaching the target group of non-psychology students via posters and notices at the University of Bergen’s different faculties and via social media platforms. The primary inclusion criterion was age ranging from 19 to 29 years old. The primary exclusion criterion was studying psychology, as psychology students are expected to possess knowledge about the methods involved, which could compromise their status as naïve participants. In total, 40 participants (20 men and 20 women) met the inclusion criteria. Of them, 39 completed the experiment. Four participants were excluded before conducting the statistical analyses, as they did not meet the protocol requirements. The latter included successful awakenings on a minimum of 5 out of 6 nocturnal awakenings, and at least 30 min of wakefulness across the awakenings as recorded by online-questionnaires. The final sample comprised 35 adults (16 men and 19 women) ranging from 19 to 26 years old (mean age 21.8). All participants were, as assessed by self-report, mentally and physically well. They had no history of pain-related conditions, or other chronic illnesses, and they were not regularly taking prescription medicine. Each participant received 500 NOK (approximately 60 USD) at the end of the study as compensation.

The obtained sample size was deemed sufficient when setting the power to 0.80, alpha to 0.05 (two-tailed), having at least three repeated assessments for each dependent measure (with an inter-correlation of 0.30) and setting the effect size (*d* = 0.50) to medium (Hedeker et al., 1999, pp. 70–93). In the case of multiple comparisons Bonferroni adjustment will be used in order not to capitalize on chance.

### Experimental Protocol

**FIGURE 1:**
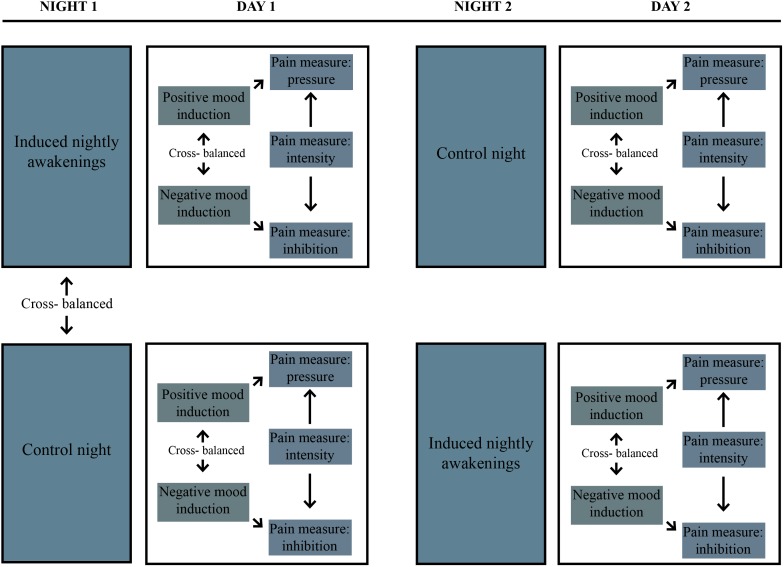
Overview of the experimental protocol used.

Figure 1 provides an overview of the experimental protocol. An experimental counterbalanced crossover design was used, where the participants were subject to an experiment-night including sleep fragmentation, and a control-night of undisturbed sleep. The total experimental period was 14 days, where sleep was subjectively assessed through filling out a sleep diary every day during this period. The two conditions, induced nightly awakenings (fragmented sleep) vs. a control night (experimentally undisturbed sleep), were separated by 6 days of normal sleep, and the order of the two conditions were randomized using www.randomizer.org. The participants were not informed of the order of the experiment-night and the control-night until the day before.

Assessment of pain during daytime followed both sleep conditions. All participants were subjected to pain tests on the morning following both the night of fragmented sleep and the control night. All participants were tested at the same time of day, on both assessment days. The participants were also introduced to a second independent variable on the assessment days; mood induction, comprising positive and negative mood induction, respectively. The different moods were induced before the pain assessment; three different pain tests were subsequently performed. The participants were instructed to sleep as usual in the last 5 days before the experiment and the control night. Their sleep amount was verified using a sleep diary. On the experiment-night and the control night, the participants were instructed to go to bed at 22.30, and to spend at least 8 h in bed. The participants were instructed not to drink alcohol, and to discontinue any use of analgesics for at least 24 h before the assessment days.

#### Sleep Fragmentation

A forced awakening-protocol was used in order to experimentally fragment sleep. The participants were awakened by phone calls every 80 min (6 times during the night) and were at each occasion instructed to stay awake for at least 10 min. In order to confirm these awakenings, the participants were instructed to complete online questionnaires, and to fill out sleep diaries the following morning. The participants were instructed to complete a series of unrelated online questionnaires, designed to take at least 10 min to complete during each nightly awakening. The online questionnaires were created using the website www.classmarker.com, which recorded the participant’s answers, as well as the time of night and duration of their activity on the website, hereby confirming adherence to protocol. The mean wakeful time as recorded by the website, was 50 min (min. = 0:30, max. = 1:46, *SD* = 0:15). Out of the nightly phone calls, across 35 participants, 209 out of 210 were successful in waking up the participants.

#### Karolinska Sleepiness Scale (KSS)

To document the participants’ subjective sleepiness after both the experiment and control night, the KSS (Kaida et al., 2006, pp. 1574–1581) was administered. The KSS has one item, where the participants rate current experienced sleepiness on a scale ranging from 1 (very awake) to 9 (very sleepy, it is strenuous to stay awake).

#### Sleep Diary

The participants completed a sleep diary upon awakening, every morning throughout the entire experimental period. A sleep diary is a subjective assessment of different sleep parameters, kept from day to day. The variables derived from the sleep diary include sleep onset latency, wake after sleep onset, sleep efficiency, total sleep time, and assessment of sleep quality (Grønli et al., 2016).

#### Mood Induction

On each of the assessment test days (following both sleep fragmentation and normal sleep), the participants were induced to both a positive (joy), and a negative (sadness) mood, in a counter-balanced order. Thus, each participant underwent a total of four mood-inducing sessions throughout the experiment. Evidence suggests that exposure to clips is the most suitable method when inducing mood states such as joy, anger, disgust, fear, and surprise (Gross and Levenson, 1995, pp. 87–108). In line with Rottenberg et al. (2007, pp. 9–28), the participants were consequently shown selected film clips which have been found to reliably induce specific mood states (Gilet, 2008, pp. 233–239). In order to induce positive mood, participants were shown a clip from the film “When Harry Met Sally”, lasting 2 min and 35 s, and a clip from a filmed stand-up-comedy routine starring Bill Cosby lasting 2 min and 1 s. To induce negative mood, the participants were shown a film clip from “The Lion King,” lasting 2 min and 11 s, and “The Champ,” lasting 2 min and 51 s. Facial feedback was used to enhance the mood induction as, in accordance with facial feedback theory, mood or emotion can be elicited by activating muscles involved in facial expressions (e.g., smiling or frowning) (Ekman et al., 1972). The participants were, whilst being subjected to positive mood-induction, instructed to hold a pen using their teeth only, (in accordance to Strack et al., 1988, pp. 768–777) hereby activating/facilitating the facial muscles that are a part of the smiling response (zygomaticus major or the risorius muscle) (Strack et al., 1988, pp. 768–777). Whilst participants were subjected to negative mood induction, they were instructed to hold a pen using their lips only, hereby likely contracting the orbicularis oris muscle that would be “incompatible with contracting the muscles used in smiling” (Strack et al., 1988, p. 770). In order to measure emotion elicitation, and as such to validate the mood induction procedure, a shortened version of the Emotion Self-Report Inventory by Gross and Levenson (1995, pp. 87–108), using a 10 cm visual analog scale was administered. Here, the participants’ levels of the eight main emotional states (amusement, anger, contentment, disgust, fear, neutral, sadness, and surprise) were registered (Gross and Levenson, 1995, pp. 87–108).

#### Pain Tests

The effect of mood induction on pain and pain inhibition was measured by combining two pain tests; pressure pain and a CPT. Pressure pain was assessed by a hand-held pressure algometer (Wagner FDIX/50, Wagner, United States) with a circular probe of 1 cm^2^. Pressure was applied to the palm of the participant’s’ dominant hand at a steady increasing rate. The test was conducted three times and was discontinued when the participants described the pressure as becoming painful. The maximum pressure was recorded, and the mean value of these three assessments was included as a measure of perceived pressure pain threshold (PPT) (Neziri et al., 2011, pp. 376–383).

The CPT was performed by the use of a bath of circulating cold water of 2 degrees Celsius (Julabo FP40-HE; part no. 9212640). During the CPT, the participants submerged their dominant hand and wrist into the water and kept it there as long as they were able to, up to a maximum of 120 s (2 min). Thermal pain simulation is a commonly used method to induce pain experimentally (Naidu et al., 2011, pp. 632–637) and the procedure is considered to have high reliability and validity (e.g., Mitchell et al., 2004, pp. 233–237).

Three painful stimuli were delivered. Firstly, the PPT was measured. Secondly, the CPT was performed. Thirdly, the pressure pain test was repeated immediately following the CPT, to test pain inhibition. The painful CPT is expected to cause a transient pain inhibitory effect on subsequent pain stimuli. This pain-inhibiting-pain paradigm is denoted CPM (Yarnitsky et al., 2010). Since the CPT induces a transient pain inhibiting effect, the participants waited a minimum of 30 min before being subjected to the CPT again, in a bid to increase the likelihood of the pain inhibiting effect ceasing.

Subjective reports of perceived pain intensity during the CPT were provided on a Verbal Numerical Rating Scale where 0 was equivalent to feeling no pain, and 10 was equivalent to the worst pain imaginable. Participants were asked to rate their pain after 4 s, and subsequently every 9 s thereafter until the hand was withdrawn (Johansen et al., 2014, pp. 341–348). Two variables were derived from the CPT: Cold pain tolerance (the number of seconds the participants held their hand in the water before withdrawal) and cold pain intensity (pain evaluation on the Verbal Numerical Rating Scale whilst the participants had their hand submerged).

### Ethical Considerations

The Regional Committee for Medical and Health Related Research (Health Region West) approved the study protocol (no. 2016/28/REKVEST). Written, informed consent was obtained from all participants before entering the study.

### Statistical Analyses

All analyses were performed using SPSS version 24. Firstly, proof of manipulation was performed. A paired sample *t*-test was used to assess whether successful sleep fragmentation was achieved, by comparing the sleep diary variables the sleep fragmented night with the control night and the KSS-scores following the sleep fragmentation night, with the scores after the control night. Mood status following positive and negative mood induction was assessed by a paired sample *t*-test comparing the sum of scores on the positive emotions (amusement and contentment) and negative emotions (anger, sadness, contentment, and fear) of the Emotion Self-Report Inventory across conditions. To be considered successful, positive emotions had to be significantly higher than negative emotions for the positive mood condition, while negative emotions had to be significantly higher than positive emotions for the negative mood condition. The effects of the experiment were analyzed by linear mixed models. A restricted maximum likelihood approach was used as this produces unbiased estimates of variance and covariance parameters. Sleep (undisturbed vs. fragmented), induced emotion (positive vs. negative), and time (1–13 for CPT and 1–3 for pain pressure) comprised the fixed factors. For cold pain tolerance and pain inhibition, time was a constant, and was thus not included in the model. Pain inhibition was operationalized as the mean pressure value of the pressure test before the CPT, minus the mean pressure value after the CPT. If perceived threshold was higher after the CPT this suggests that pain inhibition had occurred and corresponds to negative values. Random intercept was included in all models (Harville, 1977; West et al., 2014). In all, a total of four dependent pain measures were included: cold pain intensity and cold pain tolerance, perceived PPT and pressure pain inhibition.

## Results

### Manipulation Checks

The sleep fragmentation night led to significantly worse subjective sleep and higher subjective sleepiness the following morning than the control night and subsequent morning. According to the sleep diaries, participants experienced significantly more wake time after sleep onset on the sleep fragmentation night, compared to the control night. On the sleep fragmentation night, they experienced significantly more wake time after sleep onset [(*m* = 74.1 min, *SD* = 15.8) vs. (*m* = 8.5 min, *SD* = 12.9), *p* < 0.01, *d* = 4.55], a higher number of awakenings [(*m* = 6, *SD* = 0.2) vs. (*m* = 0.9, *SD* = 1.5), *p* < 0.01, *d* = 4.77], significantly less total sleep time [(*m* = 7.0 h, *SD* = 0.8) vs. (*m* = 8.0 h, *SD* = 0.9), *p* < 0.01, *d* = −1.17], significantly worse sleep efficiency [(*m* = 78.5%, *SD* = 8.2) vs. (*m* = 91.5%, *SD* = 7.6), *p* < 0.01, *d* = −1.64], and significantly worse subjective sleep [(*m* = 1.9, *SD* = 0.9) vs. (*m* = 3.7, *SD* = 0.9), on a scale from 0 to 5, *p* < 0.01, *d* = −2.00] compared to the control night. There was no difference in time spent in bed (*m* = 8.9 h vs. 8.7 h). The participants also reported feeling significantly more tired the morning after sleep fragmentation, as indicated by the KSS-scores [(*m* = 5.5, *SD* = 1.9) vs. (*m* = 3.8, *SD* = 1.8), *p* < 0.01, *d* = 0.92]. In the negative mood induction condition, significantly higher scores on the negative emotions compared to the positive emotions were found [(*m* = 12.6, *SD* = 9.8) vs. (*m* = 7.0, *SD* = 8.4), *p* < 0.01, *d* = 0.61]. In the positive mood-induction-condition, significantly higher scores on the positive than the negative emotions were reported [(*m* = 19.2, *SD* = 6.8) vs. (*m* = 12.8, *SD* = 7.8), *p* < 0.01, *d* = 0.87].

### Cold Pain Tolerance

There was no main effect of Sleep (*F*_1,136_ = 0.16, *p* > 0.05) or Induced emotion (*F*_1,136_ = 0.02, *p* > 0.05) on cold pain tolerance. Further, the two-way interaction Sleep × Induced Emotion was not significant (*F*_1,136_ = 0.00, *p* > 0.05).

### Cold Pain Intensity

A main effect of Sleep was found (*F*_1,1768_ = 9.84, *p* < 0.01). The mean pain rating in the undisturbed vs. the sleep fragmented condition was 8.24 (*SD* = 2.2) and 8.45 (*SD* = 2.2), respectively, (*d* = −0.10).

The main effect of Induced emotion was not significant (*F*_1,1768_ = 1.73, *p* > 0.05). The main effect of time was significant (*F*_12,1768_ = 229.44, *p* < 0.01). The pain rating at time 1 [(*m* = 3.42, *SD* = 2.07), (*d* = −3.56)], time 2 [(*m* = 5.99, *SD* = 1.98), (*d* = 2.08)], time 3 [(*m* = 7.51, *SD* = 1.78), (*d* = −1.23)] and time 4 [(*m* = 8.39, *SD* = 1.51), (*d* = −0.71)], were all significantly lower (Bonferroni; all *p* < 0.01) than at time 13 (*m* = 9.34, *SD* = 1.12), which constituted the contrast.

The Sleep × Induced emotion interaction (*F*_1,1768_ = 0.60, *p* > 0.05), the Sleep × Time interaction (*F*_1,1768_ = 0.04, *p* > 0.05), the Induced emotion × Time interaction (*F*_1,1768_ = 0.03, *p* > 0.05) and the Sleep condition × Induced emotion × Time interaction (*F*_1,1768_ = 0.04, *p* > 0.05) were all non-significant.

### Perceived Pressure Pain Threshold

There were no main effects neither for Sleep (*F*_1,406_ = 1.20, *p* > 0.05), Induced emotion (*F*_1,406_ = 1.81, *p* > 0.05) nor of Time (*F*_1,406_ = 1.34, *p* > 0.05) on perceived PPT. Further, none of the two-way interactions Sleep × Induced emotion (*F*_1,406_ = 0.02, *p* > 0.05), Sleep × Time (*F*_1,406_ = 0.03, *p* > 0.05) and Induced emotion × Time (*F*_1,406_ = 0.53, *p* > 0.05) were significant. The three-way interaction Sleep × Induced emotion × Time (*F*_1,406_ = 0.10, *p* > 0.05) was not significant.

### Pressure Pain Inhibition

A main effect of Sleep was not found (*F*_1,136_ = 2.07, *p* > 0.05) showing no difference in pressure pain inhibition in the sleep fragmented (*m* = −3.42 newton, *SD* = 8.85), compared to the undisturbed sleep condition (*m* = −5.98 newton, *SD* = 11.83), (*d* = 0.40). No main effect of Induced emotion (*F*_1,136_ = 0.00, *p* > 0.05) was found. The Sleep × Induced emotion (*F*_1,136_ = 0.03, *p* > 0.05) interaction was significant.

## Discussion

The present study explored the interaction between sleep fragmentation and positive and negative mood on pain perception. Firstly, this study reports increased cold pain intensity in healthy young adults after one night of experimentally induced sleep fragmentation. This is in accordance with relevant current research findings both after total and partial sleep deprivation (Finan et al., 2013, pp. 1539–1552), and after sleep fragmentation (Iacovides et al., 2017, pp. 844–854), and thus supports the adverse effect of disrupted sleep on pain perception. Surprisingly, this study found no significant results of sleep fragmentation with regards to cold pain tolerance, which is not in line with parallel research (e.g., Smith et al., 2007; Iacovides et al., 2017). Furthermore, there was no difference in the effect of positive or negative mood on pain, and affect was not found to be a moderator in the sleep–pain relationship. Lastly, a time effect of the pain intensity was found showing a lower pain rating in the beginning, compared to the end of the CPT. This finding is in general agreement with standard responses to the CPT (Zheng et al., 2014). Despite successfully fragmenting the participant’s sleep, as confirmed by the manipulation checks conducted, only cold pain intensity was affected. In comparison, Iacovides et al. (2017) reported that young healthy participants that were subject to one night of experimental sleep fragmentation, reported increased deep muscle and superficial pain sensitivity. They also reported that this effect was reinforced by a second night of sleep fragmentation. In the present study, sleep was manipulated for one single night. Thus, it is conceivable that the effect would have been greater on all the measures if the participants sleep had been disturbed, as previously demonstrated for two nights (Iacovides et al., 2017, pp. 844–854) or more (Onen et al., 2001, pp. 35–42). The cumulative effect of time should consequently be investigated in future studies. The fact that not all pain outcomes turned out significant in this study, may also reflect that the included outcome measures varies in terms of sensitivity. It may also reflect differences in statistical power, as some outcomes were based on one single measure in each condition (e.g., cold pain tolerance), whereas other measures consisted of several measures in each condition (e.g., cold pain intensity). Another explanation for the somewhat divergent effects, is that the different outcomes assess different aspects of pain (e.g., tolerance, intensity, threshold, and inhibition) which may be affected differently by sleep fragmentation. Thus, this may also reflect the complexity of pain perception in relation to sleep deprivation.

Despite successful induction of positive and negative mood, as confirmed by the manipulation check, there were no significant effects of induced emotion on pain in the present study. Although some criticism has been expressed regarding the effects of facial feedback (Wagenmakers et al., 2016, pp. 917–928), the use of film clips, as employed in the present study, has been shown to be a reliable way to induce emotions (Gross and Levenson, 1995, pp. 87–108). An explanation for the lack of effects of induced emotion on pain may be that emotions have a weaker effect on pain than on sleep. This assumption is in line with a recent study of how pain patients attribute the reciprocal impact of pain, sleep and mood (Blågestad et al., 2016, p. 1689). However, the strength of manipulations of the two independent variables is difficult to compare, and another explanation of the lack of effects of induced emotions, may be that larger differences in mood than those obtained in the present study, need to be present in order to detect differences between conditions in terms of nociception.

Previous studies have shown that negative mood in some cases mediates the effects of poor sleep on pain perception (O’Brien et al., 2010, pp. 310–319). It has therefore been suggested that mood also may moderate the impact of sleep on pain perception. However, in the present study none of the sleep x induced emotion interaction effects were significant, hereby implying that affect does not in fact moderate the sleep–pain relationship. This finding is in accordance with the results from a study conducted on pain perception in children, finding support for the notion that affect mediates, but does not moderate the sleep–pain relationship (Evans et al., 2017, pp. 1087–1095). However, seeing as this study utilized one single night of sleep fragmentation on healthy, young adults, it is conceivable that generalizability might be challenged in terms of the cumulative load one could expect chronic pain patients to experience.

Several mechanisms may be in play concerning the pain amplifying effect of sleep fragmentation. The dopaminergic network of mesolimbic and nigrostriatal circuity, is strongly related to pursuing pleasure and may be deactivated through receptor down-regulation following sleep loss, and may consequently affect pain perception (Finan et al., 2013, pp. 1539–1552). It has further been hypothesized that sleep loss modifies the opioid receptor function (Fadda et al., 1992, pp. 153S–156S). Sleep loss has further been shown to increase inflammatory cytokines, such as interleukin-6, which seems to influence nociception (Heffner et al., 2011, pp. 35–41; Haack and Mullington, 2005, pp. 56–64.). Future studies should investigate whether some of the aforementioned mechanisms may be underlying the observed effects of the present study.

### Strengths and Limitations

There are some limitations regarding the present study that should be noted. The sample was recruited using convenience-sampling. This kind of sample usually increases the risk of biases. However, as an experimental counter-balanced design was used, it is not conceivable that sample characteristics influenced the findings. Further, as basic pain mechanisms were studied, the results are arguably generalizable to other populations, although utilizing only a single night of fragmented sleep as is done in this study, potentially challenges the generalizability to a chronic pain population, which one generally can expect to experience more chronic sleep disturbances. Although the CPT is regarded as an effective tool in simulating the effects of chronic pain afflictions (Mitchell et al., 2004, pp. 233–237), the procedure was repeated four times for each participant. Hence, it cannot be ruled out that some participants developed coping strategies followed by a feeling of control (Koolhaas et al., 2011, pp. 1291–1301). This repetition of the CPT-procedure leads to the possibility of a learning-effect in the participants, in that they may have experienced that the CPT was terminated after 2 min, which in turn may have influenced the results, more than the actual experienced pain and the effect of sleep on this pain experience. Motivational changes were not measured in the present study and can therefore not be ruled out as a potential influence. As the participants held their hand submerged for a variable duration of time during the CPT, this might have influenced the degree of pain inhibition assessed immediately thereafter. Still, a significant effect of sleep on pain inhibition was found, making it less likely that the variable time submerged acted as a confounding variable. As it is impossible to blind participants to sleep condition, it further cannot be ruled out that the results may have been influenced by demand characteristics as participant could have figured out the assumptions (poor sleep increases pain perception) underlying the present study (Orne, 1962, pp. 776–783). The present study utilized sleep diaries as a subjective assessment of sleep. The use of subjective data always increases the risk of biases. However, one study investigating how well self-reports reflect objective measures of sleep in terms of sleep duration, found a moderate correlation (0.45) between the objective measures of actigraphy, and the subjective measures of sleep diaries (Lauderdale et al., 2008). One study found that in conjunction with a sleep diary, the accuracy of actigraphy is significantly improved (Horne and Biggs, 2013).

An obvious asset of the present study is the use of fragmented sleep, as opposed to total sleep deprivation, as it has been shown that the former is the best way to mimic the sleep patterns of individuals suffering from chronic pain (e.g., Finan et al., 2013, pp. 1539–1552). As such, this contributes to the ecological validity of the present study, in terms of relevance for chronic pain patients. A further strength of the present study entails the counter-balanced experimental procedure, ensuring high internal validity. Further, sleep was altered by sleep fragmentation rather than by total/partial sleep deprivation as the former is regarded as a more ecologically valid model to study the sleep–chronic pain relationship than the latter (Goodin et al., 2011, pp. 913–922; Iacovides et al., 2017, pp. 844–854). The present study is moreover one of the very first to address the potential moderating effect of affect in relation to the sleep–pain relationship. Finally, it should be noted that testing was performed at the same time in all conditions, limiting differential influences from circadian factors on pain perception (Aviram et al., 2015, pp. 1137–1144).

## Conclusion

The present study suggests that even one night of fragmented sleep has a negative impact on the perception of pain intensity, but not pain tolerance or pain inhibition. Inducing negative and positive emotions did not seem to moderate the sleep–pain relationship. The mechanisms by which sleep disruption affects pain sensitivity are still largely unknown, which underlines the need for further research.

## Ethics Statement

This study was carried out in accordance with the recommendations of the guidelines of the Regional Committee for Medical and Health Related Research (Health Region West), with written informed consent from all subjects. All subjects gave written informed consent in accordance with the Declaration of Helsinki. The experimental protocol was approved by The Regional Committee for Medical and Health Related Research (Health Region West), with no. 2016/28/REK VEST.

## Author Contributions

RR contributed to designing the study, recruited the participants in the study, collected the data, and also contributed in analyzing the data and wrote the manuscript. TB supervised the project, contributed to designing the study, and the theory development. And also contributed to data collection, analysis and critically reviewed the manuscript. SP supervised the project and contributed to designing the study and the theory development. He also contributed in the choice of analysis, by analyzing data, and by critically reviewing the manuscript. IN contributed to designing the study, the theory development, and critically reviewed the manuscript. DM contributed to the theory development. He also contributed in deciding the methods used, and by critically reviewing the manuscript. All authors have approved of the final version of the manuscript to be published.

## Conflict of Interest Statement

The authors declare that the research was conducted in the absence of any commercial or financial relationships that could be construed as a potential conflict of interest.
